# Comparative Analyses Suggest Genome Stability and Plasticity in *Stenotrophomonas maltophilia*

**DOI:** 10.3390/ijms262110477

**Published:** 2025-10-28

**Authors:** Danny Khar Chen Sum, Yee Yee Chong, Joon Liang Tan

**Affiliations:** 1 Faculty of Information Science and Technology, Multimedia University, Melaka 75450, Malaysia; 2 Center for Intelligent Cloud Computing, CoE for Advanced Cloud, Multimedia University, Melaka 75450, Malaysia

**Keywords:** mobile genetic elements, comparative genomics, genome stability

## Abstract

*Stenotrophomonas maltophilia* (*S. maltophilia*) is a multidrug-resistant opportunistic pathogen. There are an increasing number of case reports on *S. maltophilia* infections in recent years, and the species is becoming a public health concern. Many studies have focused on profiling and pangenome of the species, particularly on their antibiotic resistance and virulence genes. However, there is a lack of studies on mobile genetic elements (MGEs), a subset of pangenome that significantly contributes to the diversity, stability, and plasticity of a population. In this study, 20 genomes of *S. maltophilia* were downloaded from the NCBI Genome database. The genomes were subjected to profiling of MGEs, their impact on the population structures, and the evaluation of evolutionary trends of the core genomes. The cataloguing of MGEs indicated active horizontal gene transfer events in the *S. maltophilia*’s population. Multiple virulence and drug resistance genes were predicted within and outside of the MGEs. We observed multiple chromosomal rearrangements in the genomes, most likely caused by MGEs, affecting up to approximately 50% of a single genome sequence. A high number of linkage disequilibrium sites were also predicted in the core genomes. This study provides insights into stability in the core and plasticity in the accessory regions in the *S. maltophilia* population.

## 1. Introduction

*Stenotrophomonas maltophilia* has emerged as a global opportunistic pathogen that poses a significant threat in both nosocomial and community-acquired settings [1]. It has been recognized as an important multidrug-resistant pathogen that primarily infects immunocompromised patients. Infections are frequently reported in patients with underlying conditions such as cystic fibrosis, hematological malignancies, as well as in neonates and individuals undergoing prolonged hospitalization [2,3]. The clinical manifestations are diverse, including bacteraemia, sepsis, and soft tissue infections [4,5]. The clinical challenges posed by *S. maltophilia* are underscored by its association with high morbidity and mortality rates, which can range from 37.5% to 56% in patients with bacteremia [6,7].

The pathogenic success of *S. maltophilia* is linked to its dual existence as both a clinical threat and a ubiquitous environmental bacterium. The species is frequently isolated from a vast array of natural and anthropogenic habitats like soil, plant rhizospheres, and aquatic environments [8,9]. This environmental ubiquity provides a persistent reservoir from which the bacterium can be transmitted into healthcare settings. The capacity for biofilm formation enables *S. maltophilia* to colonize abiotic surfaces such as catheters and endoscopes, which become the critical vectors for nosocomial transmission [2,10]. Any strains from environments may possess the foundational toolkits required for opportunistic pathogenesis. Comparative genomic studies have shown that the distinction between clinical and environmental isolates is not phylogenetically defined, as strains from both niches often cluster together and share a substantial portion of their genomic content [11,12]. Thus, the transition from an environmental isolate into a clinical pathogen is not a result of speciation into a virulent lineage, but it is facilitated by the organism’s genomic flexibility.

With the advancement of whole-genome sequencing, it has been revealed that the genetic repertoire of a species is far greater than that contained within any single isolate. This concept was formalized as the “pangenome”, which represents the entire set of genes found across all strains of a species [13]. Multiple genomic studies have demonstrated that *S. maltophilia* possesses a large and open pangenome [14,15]. For an opportunistic pathogen like *S. maltophilia*, the open pangenome enables the species to adapt between environmental and clinical settings. Its accessory genome allows environmental strains to acquire genes for traits like antibiotic resistance or virulence, thereby becoming successful opportunistic pathogens. Therefore, it is vital to understand the mechanisms that shape this dynamic accessory genome and decipher its pathogenic potential.

The primary evolutionary force that expands the accessory genome and creates an open pangenome structure is horizontal gene transfer (HGT) [16]. HGT is the acquisition of genetic material from non-parental lineages. Bacteria can acquire complex traits encoded by clusters of genes, such as antimicrobial resistance, virulence, and metabolic capabilities, through HGT. This helps to accelerate their adaptation to new environmental niches and selective pressures far more rapidly than is possible through vertical inheritance and point mutation alone [16]. This is primarily mediated by mobile genetic elements (MGEs). MGEs are found to be the principal vectors responsible for the dissemination of antibiotic resistance genes among clinically relevant pathogens [17]. Different classes of MGEs play distinct but synergistic roles in this process. The classes of MGEs are plasmids, phages, transposons, insertion sequences (ISs), and integrative and conjugative elements (ICEs) [18]. Plasmids can transfer multidrug-resistance cassettes between bacterial hosts, while transposons and ISs can mobilize antibiotic-resistant genes from a chromosome to a plasmid or vice versa and facilitate their subsequent spread. ICEs are large, “mosaic” MGEs that typically reside integrated within a host chromosome but can excise to form a circular intermediate and transfer to a new host via a self-encoded conjugation system [19]. ICEs are powerful drivers of evolution that are capable of mobilizing large genomic islands that can carry arrays of genes conferring antibiotic resistance, novel metabolic capabilities, or virulence factors in a single transfer event. Recently, characterization of a 91 kb clc-type ICE in an *S. maltophilia* isolate was observed to carry multiple resistance genes, including *sul1*, *blaVIM-1*, and *aac(6′)-Ib.* This serves as strong evidence that MGEs can introduce a suite of resistance mechanisms into this opportunistic pathogen in a single HGT event [20]. Bacteriophages (phages) can integrate their DNA into the host chromosome as prophages through their lysogenic life cycle. During this process, they can mediate the transfer of bacterial genes from one host to another via transduction [21]. Prophages themselves frequently carry cargo genes like virulence factors, which alter the host’s phenotype in a process known as lysogenic conversion [22].

ISs and transposons are the simplest forms of MGEs [23]. They can move nearby genes and also alter the genome structure by triggering large-scale genomic rearrangement events such as inversion, deletion and translocation [24,25]. These MGEs act in a system. Their combined action can cause a wide range of genetic changes, from adding genes to reorganizing the chromosome. These elements facilitate the HGT that allows for the rapid acquisition and dissemination of novel genetic materials between bacterial cells, although of different species. This dynamic interplay between MGEs and their genetic cargo underscores the necessity of analyzing the mobilome to understand the emergence and spread of MDR bacteria.

The activity of the mobilome creates a central paradox in bacterial evolution. However, if the genome is constantly under threat of disruption by MGEs, the species will have issues maintaining its functional integrity and evolutionary coherence. The balancing of the two aspects can be achieved from two sets of opposing forces, which are the disruptive forces of HGT and the conservative forces of selection and host defense that promote stability. The forces are reflected in the core and accessory genome of bacteria. The core genome is not as flexible as the accessory genome. Purifying selection [26] protects the conservation of essential genes and removes harmful changes in the hosts. This evolutionary pressure ensures the stability of the core functions that define a species. One of the key statistical signatures of this stability is by evaluating linkage disequilibrium (LD) [27], which is the non-random association of gene variants at different locations in the genome. Certain combinations of variants across the core genome are inherited together more often than expected by chance. The presence of LD in the core genome is a powerful indicator of selective forces that resist genomic disruption and maintain the essential genes despite the presence of MGEs [27]. A clear example is seen in *Listeria monocytogenes*, where a whole-genome analysis revealed strong LD [28]. This pattern reflects its clonal genetic structure and low rates of recombination, suggesting a limited exchange of foreign genetic material. The practical impact of studying these patterns is significant, as analyzing LD allows scientists to map disease-associated genes, infer the intensity of natural selection, and estimate the age of mutations. On top of LD, bacteria have evolved diverse defense systems against invading MGEs to achieve stability. Restriction-modification (RM), CRISPR-Cas and Toxin-Antitoxin (TA) are the most commonly reported defense systems in the bacteria [29]. RM functions as an innate immune system that uses methylation to distinguish the host’s own DNA from foreign DNA and destroy the latter [30]. CRISPR-Cas system acts as an adaptive immune system that captures small pieces of DNA from invaders and stores them as memory, thereby allowing the host to recognize and destroy the invaders [31]. TA system plays dual roles where they can trigger processes like abortive infection [32] that cause a cell to self-destruct to prevent further hazards caused by viruses. TA systems are often carried on MGEs to ensure the MGEs are not lost during cell division by killing any daughter cells that failed to inherit them [33].

*Stenotrophomonas maltophilia* is known to possess a large and open pangenome. It is a hallmark of a species that engages in extensive HGT and exhibits significant genomic diversity [14]. This plasticity is evident, as there is substantial variation in the genome arrangement and gene content observed among closely related isolates [34]. Despite this recognition, the precise roles of MGEs are not yet fully elucidated. A systematic investigation is needed to investigate the MGEs within the *S. maltophilia* complex and to understand how their activity contributes to the organism’s evolution from a ubiquitous environmental bacterium into a challenging clinical pathogen. Therefore, we aimed to understand the MGEs in *S. maltophilia* and the potential impact of recombination events on the evolution of the species.

## 2. Results

### 2.1. Overview of Stenotrophomonas maltophilia

The query searched in the Refseq database resulted in twenty *S. maltophilia* genomes being selected and downloaded for this study. The genome sizes ranged from 4,202,951 bp to 5,086,181 bp. The minimum and maximum numbers of protein-coding genes were 3707 and 4692, respectively. The downloaded genomes were grouped based on clinically and non-clinically isolated strains (Table 1).

### 2.2. Distribution of Mobile Genetic Elements(MGEs)

#### 2.2.1. Integrative and Conjugative Elements (ICEs) and Integrative and Mobilizable Elements (IMEs)

There were ten strains predicted with T4SS-type ICE, and their lengths range from 19,559 bp to 51,856 bp. The minimum and maximum GC contents were 59.95% and 67.06%, respectively, with an average of 64.02%. Five genomes contained a single ICE, four contained two ICEs, and one genome contained three. All the T4SS-type ICEs were classified as either typeG or typeT mating pair formation systems. Ten out of sixteen ICE were predicted to be either from Tn4371 and ICEclc families, where the family of six ICEs was not classified into any of the families in the ICEBERG database. Annotation of all the predicted ICE regions indicated the presence of a single T4CP2 protein. A homology search against the database showed high sequence similarities with other *Stenotrophomonas* species instead of *S. maltophilia*. However, most sequences consistently showed higher similarities to proteins from *Pseudomonas aeruginosa*.

A total of 21 IMEs were predicted across 14 *S. maltophilia* genomes. The sizes of IMEs range from 6421 bp to 35,310 bp. Compared to ICE, IMEs showed a lower range of GC compositions from 55.08% to 61.96%, with an average of 58.94%. Three genomes had a maximum of three IMEs, one genome with two IMEs, and ten genomes with a single IME (Table S1).

#### 2.2.2. Prophages

**Figure 1:**
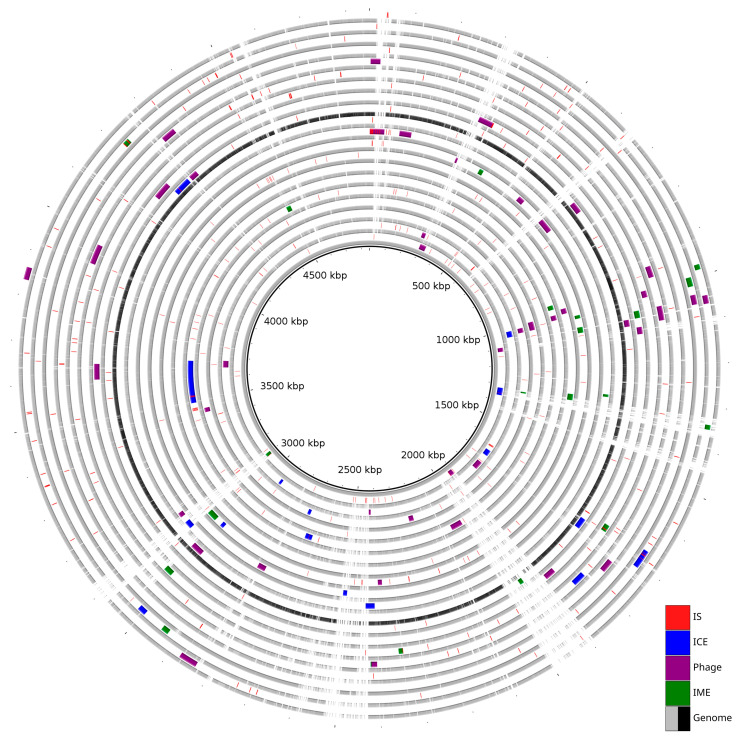
Distribution of Mobilome across 20 genomes. The innermost grey ring represents the reference genome (strain K279a). The subsequent rings alternately represent a query genome and its predicted MGEs.

Prophages were identified in all analyzed genomes except in *S. maltophilia* strain KMM 349. In total, intact prophages were predicted in nineteen strains, and incomplete prophages were only present in eight. The number of prophages per genome ranged from one to five. The sizes of intact prophages ranged from 6.6 kbp to 51.7 kbp, while incomplete prophages were smaller, ranging from 3.4 kbp to 30.8 kbp. The average GC content of all predicted prophages was approximately 65%, consistent with the host genomes’ GC content of 65–66% (Table S2). The analysis showed the presence of common and strain-specific prophages. The most prevalent intact prophages were PHAGE_Escher_vB_EcoM_ECOO78 and PHAGE_Escher_vB_EcoM_ECO1230_10, each identified in seven different genomes. In contrast, eleven prophages were unique to a single genome, including PHAGE_Salmon_epsilon15 in strain JUNP497 and PHAGE_Synech_S_CBS1 in strain K279a. Multiple prophages were observed to be localized at position 1 mbp with reference to the K279a genome. A summary of the distribution of predicted MGEs is illustrated in Figure 1.

#### 2.2.3. Insertion Sequences

Fifteen families with one possible new IS family were predicted in the *S. maltophilia* genomes used in this study (Table S3). IS21 was observed in all the *S. maltophilia* genomes. However, IS110 formed the largest number of clusters at 132, followed by IS3 at 113. Five ISs, namely IS256, IS30, IS630, IS91, and IS66, were present in a single genome. Analyses of individual genomes indicated the minimum and maximum numbers of ISs were 1 and 43, respectively. New ISs were predicted in three genomes. Multiple sequence alignment of the possible new ISs showed conservation among the sequences with more than 88% pairwise alignment similarities, which allows them to be classified within the same family.

We investigated ISs insertion sites within the genomes. Most of the ISs were inserted at intergenic regions and did not affect the structures of the hosts’ genes. Apart from hypothetical proteins, the IS were predicted upstream or downstream of functionally important genes, such as *purB*, *ompW*, *eptA*, and a few others. A small number of the ISs were also predicted to affect the hosts’ genes, e.g., truncation of several hypothetical genes and *MltA*, and at the upstream of extra copy *basR*. A few ISs were predicted to be situated neighboring to each other or with tRNAs and forming clusters at multiple sites.

**Figure 2:**
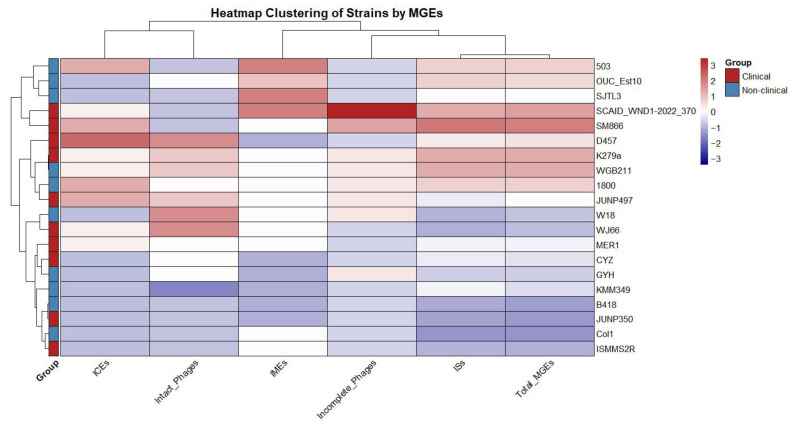
Hierarchical clustering of *S. maltophilia* strains based on the frequency of mobile genetic elements (MGEs).

There were no plasmid elements predicted in the genomes. A hierarchical clustering was performed based on the frequency of ICEs, IMEs, phages, and ISs, which grouped the 20 strains into several distinct clades based on their MGE profiles. The comparison of total MGEs between clinical (n = 10) and non-clinical strains (n = 10) showed no significant difference (U = 56, *p* = 0.68) (Figure 2), with a very small effect size (r = 0.10). This suggests that the overall MGE burden, as measured by the total count of these elements, is similar across clinical and non-clinical isolates in this dataset.

### 2.3. Genomic Rearrangements

Multiple and pairwise genome alignments showed that the genomes of the analyzed strains were largely syntenic (Figures S1–S3). Most of the conserved genomic blocks were arranged in the same order when compared to the *S. maltophilia* K279a. Despite syntenic conservation, several genomic rearrangements were observed. A translocation and inversion occurred in CYZ. The rearranged genomic regions were identified as two different prophages, PHAGE_Stenot_Smp131_NC_023588 and PHAGE_Escher_vB_EcoM_ECO1230_10_NC_027995. These prophages were not predicted to carry any antibiotic resistance or virulence factor genes. Further annotation of these regions showed that they primarily encode hypothetical proteins and phage-related proteins, such as phage tail and head proteins, capsids, lytic enzymes, and baseplate assembly proteins. A large inversion of 2,418,035 bp was identified in the pairwise genome alignment between strain K279a and SM 866 (Figures S4 and S5). The inverted region was mostly conserved between the two strains, though smaller insertions and deletions were also present. An analysis of the inverted flanking regions in strain SM 866 revealed two identical IS110 family transposases framing the inverted segment, but these transposases were absent from the corresponding region in strain K279a. No gene disruption was observed at the boundaries of the inversion when the flanking regions of strains K279a and SM 866 were compared.

A pairwise alignment of strains K279a and SJTL3 revealed an inversion associated with a Tn3 family transposase, ISPa43. An inversion was identified in the pairwise alignment between strain K279a and JUNP350. Annotation of this inverted region in both strains showed that it primarily contained genes for hypothetical proteins, and no genes known to be associated with genomic rearrangement were identified in these regions.

Translocation and inversion events were identified in the pairwise genome alignment of strains K279a and WGB211. The translocated regions were identified as ICEs containing different sets of cargo genes. The ICE in strain K279a carried genes for a virginiamycin B lyase (*vgb_2*) and a multidrug-resistance protein (*stp_1*), along with an IS630 family transposase (ISStma10). In contrast, the ICE in strain WGB211 carried genes for heavy metal resistance (*czcD_3*, *merR1_1*, *acr3_2*), a multidrug efflux pump subunit (*acrA_1*), and a type II restriction-modification (RM) system (*paeR7IM* and *paeR7IR*).

Inversion and translocation events were observed in the genome alignment between strains K279a and 503. The translocated regions were identified as ICEs. The ICE in strain 503 was found to carry only the core modules of a typical ICE, such as genes for the conjugal transfer protein and T4SS components.

### 2.4. Antibiotic Resistance Genes

The investigation of the mobilome identified ARGs within ICEs and IMEs, with no resistance determinants detected in any predicted prophage regions across the 20 genomes analyzed (Table S4). In the clinical strains, the *S. maltophilia* strain WJ66 possessed an IME containing a multi-drug resistance cassette with three ARGs identified as perfect hits to the CARD: the *sul1* gene, which confers resistance to sulfonamides through antibiotic target replacement; the *tet(C)* gene, which confers resistance to tetracycline via an antibiotic efflux mechanism; and the *aadA2* gene, which confers resistance to aminoglycosides through antibiotic inactivation. Among the non-clinical isolates, the ICEs in strains 1800 and WGB211 were predicted to carry *adeF*, associated with fluoroquinolone and tetracycline efflux, based on a strict hit with 61.08% sequence identity. In contrast, the ICE from non-clinical strain 503 contained a complex multi-drug resistance module, which included perfect hits for the *tet(C)* (tetracycline efflux) and *ANT(2*″*)-Ia* (aminoglycoside inactivation) genes, as well as five high-confidence strict hits: *APH(6)-Id* (99.64%), *APH(3*″*)-Ib* (99.63%), and *aadA3* (99.64%) for aminoglycoside inactivation; *cmlA5* (97.85%) for phenicol efflux; and *sul1* (99.64%) for sulfonamide target replacement. Notably, several of these MGE-associated genes—including the aminoglycoside resistance genes *aadA2*, *aadA3*, and *ANT(2*″*)-Ia*, the tetracycline efflux gene *tet(C)*, and the phenicol efflux gene *cmlA5*—were not identified as part of the intrinsic core resistome in any of the 20 isolates, highlighting the role of MGEs in introducing new resistance determinants into the population.

**Figure 3:**
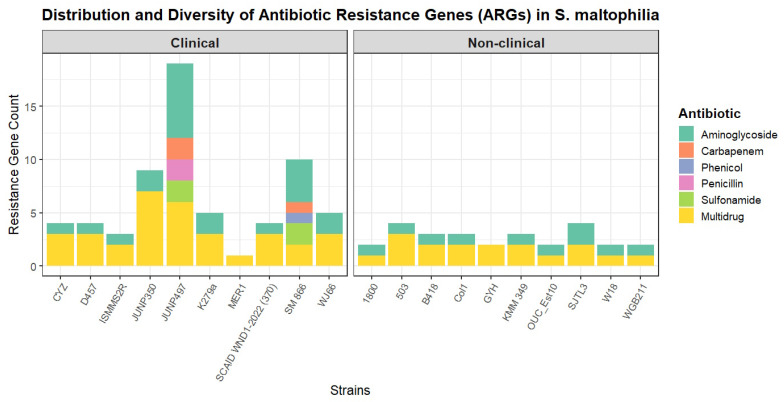
Distribution and diversity of antibiotic resistance genes (ARGs) in *S. maltophilia* strains. The stacked bar plot shows the total number and diversity of ARGs identified in each of the 20 *S. maltophilia* genomes, grouped by isolation source into clinical and non-clinical cohorts.

To assess the intrinsic resistome, a comparative analysis was performed on the genomes after the computational excision of all identified ICEs, IMEs, and phages (Table S5). The total number of ARGs was significantly higher in clinical (n = 10) compared to non-clinical strains (n = 10), a difference that was statistically significant with a large effect size (U = 83.5, *p* = 0.005, r = 0.577). However, the diversity of resistance was not significantly different, as the comparison of the number of ARG classes between the groups showed no statistical significance and only a small effect size (U = 59, *p* = 0.179, r = 0.218) (Figure 3).

### 2.5. Virulence Factors

Homology searches against the Virulence Factor Database (VFDB) identified a diverse array of putative VFs encoded within ICEs, IMEs, and phages across both clinical and non-clinical strains. These MGEs were found to carry VFs related to several key functions, including regulation, biofilm formation, adherence, and metabolism. ICEs and IMEs in both clinical and non-clinical isolates frequently harbored regulatory genes (e.g., *bfmR/S*), efflux pumps (*ade* family), and adherence factors (*upaG*) (Table S6). Prophages were also significant vectors, carrying widespread stress survival genes (e.g., *clpP*), adherence factors (*mshM*), and regulatory genes (*sigA*, *ptxR*). A list of the MGE-associated VFs, their functions, and their distribution are detailed in Table 2. The analysis of the intrinsic virulome (after removal of MGEs) showed a significant difference between the clinical and non-clinical groups (*p* = 0.034, r = 0.482). Clinical isolates were found to possess an average of 995 VFs in their core genomes, while non-clinical environmental isolates harbored a comparable average of 1026 VFs.

### 2.6. Defense-Anti-Defense and Toxin-Antitoxin (TA) System

All the *S. maltophilia* genomes were predicted with intrinsic defense and anti-defense proteins (Table S6). Twenty-six and thirty-five defense families were predicted in clinical and non-clinical genomes, respectively, of which eleven were common to both groups. There were only four anti-defense protein families, namely NADP, Anti-Pycsar, Anti-CBASS, and Anti-RM, in the current *S. maltophilia* genomes, and all were observed in both groups. The total number of anti-defense proteins was 24 and 26 in clinical and non-clinical groups. There is no statistically significant difference in the total number of defense systems between clinical and non-clinical *S. maltophilia* strains (U = 27, *p* = 0.087, r = 0.39). The comparison of anti-defense systems also showed a similar trend, that is, no significant difference between the two groups (U = 46, *p* = 0.813, r = 0.06). We further evaluated the potential of defense and anti-defense proteins for being cargo proteins of MGEs (Table S7). The analyses indicated, in addition to the proteins as described above, that further annotation of ICE and IME indicated the presence of defense proteins but without any anti-defense protein. However, annotations on the predicted prophage showed the presence of anti-defense without any defense protein. Three defense protein families (Kiwa, Shango, and AbiJ) were introduced into the *S. maltopilia* population through MGE.

TA systems were predicted in all 20 *S. maltophilia* genomes (Table S8). The number of TA systems in clinical strains ranged from 5 to 13, while in non-clinical strains, the range was 5 to 15. Clinical strains contained an average of 9.8 TA systems, and non-clinical strains had an average of 10 TA systems. The non-clinical strain OUC_Est10 contained the maximum of 15 TA systems, while the clinical strains MER1 and JUNP350 and the non-clinical strain KMM 349 contained the minimum of five TA systems each. The T10073(mazF)/AT956(vapB) and T10097(PumA)/AT10097(PumB) TA system pairs were prevalent among both clinical and non-clinical strains. Two of the predicted PumA/PumB systems were located within a prophage region and a genomic island. Additionally, the PP4151/PP4152 TA system was predicted in the clinical strain D457 and the non-clinical strain 1800. In both strains, this TA system was located within a predicted ICE. The ICE in strain 1800 also contained a predicted antibiotic efflux gene, whereas the corresponding ICE in strain D457 had no predicted virulence or resistance markers. *MazF*, *HigB,* and *Doc* were also present among the predicted TA genes.

### 2.7. Evolutionary in the Core Genomes

The pseudo-sequences from the aligned genomes as defined in Mugsy serve as a representation of the backbone for *S. maltophilia*’s population. Low recombination-over-mutation rates (rho/theta) were observed across *S. maltophilia* core genomes (0–0.03). All the genomes were predicted to have no impact to minimal impact of recombination (0–0.68), with the exception of one genome with the value of 1.03 (strain SCAID WND1-2022 (370)).

**Figure 4:**
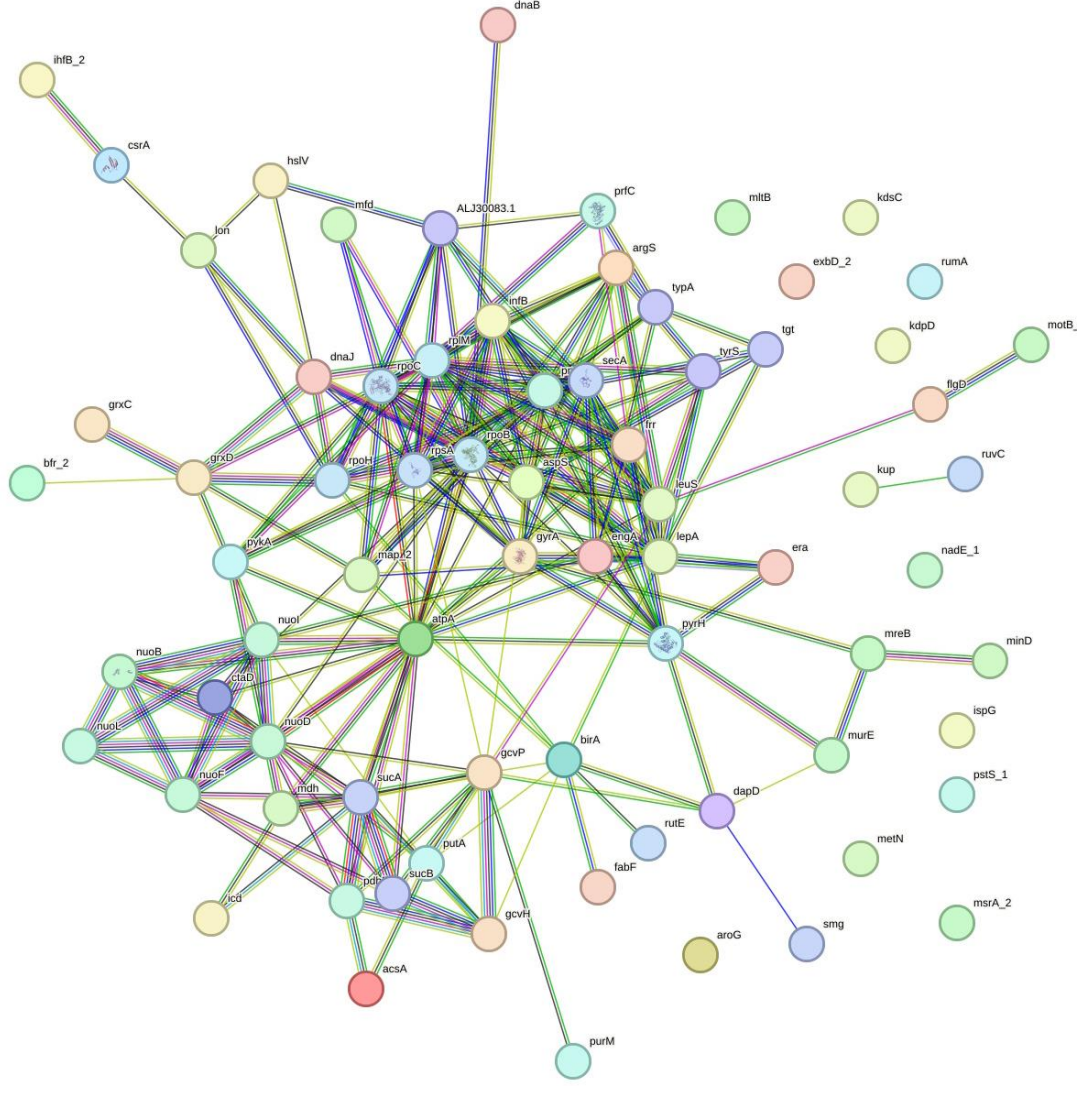
Illustration of associations of genes with linkage disequilibrium sites. Evidence (line) represented by red—fusion; green—neighborhood; blue—co-occurrence; purple—experimental; yellow—text mining; light blue—database; black—co-expression.

The observation of minimal impact and low recombination relative to the background rate of mutation raised the hypothesis of association of nucleotide variations. Further analyses indicated 3201 sites with linkage disequilibrium (LD), involving 407 genes and a single tRNA (tRNA-Leu(gag)) (Table S9). We randomly selected 20 LD sets and analyzed the corresponding genes for interactions and enrichments. All the genes from 20 respective sets showed enriched interactions. We illustrated the interactions by using one of the gene sets with LD (Figure 4). The interactions were supported by multiple pieces of evidence, represented by edges of different colors, joining the nodes that represent the genes with LD. Ten out of twenty selected LD clusters showed biological processes enrichment in regulation of translation, four in gene expression, two in translation, and one each in cell septum assembly, carboxylic acid metabolic process, cellular respiration, and ribonucleotide metabolic process. In the molecular functions, the genes were dominantly enriched in NADH dehydrogenase (quinone) activity, followed by heterocyclic compound binding, quinone binding, ion binding, carbon-nitrogen lyase activity, and purine ribonucleoside triphosphate binding. Lastly, in cellular compartments, the oxidoreductase complex was highly enriched, subsequently with Intracellular anatomical structure, catalytic complex, and oxoglutarate dehydrogenase complex. There were eleven enrichment hits in the reactome database, and all were in the infection with *Mycobacterium tuberculosis* category, with the exception of one in the latent infection category, with other responses of Mtb to phagocytosis (Table 3).

## 3. Discussion

As of June 2025, there were 27 validly published species in the *Stenotrophomonas* genus listed in the LPSN database [54]. Although multiple species are known from the genus, *S. maltophilia* still emerged as a significant species that causes public health concerns. All studies on *S. maltophilia* highlighted its multidrug resistance and pathogenicity, irrespective of its lineages, geographical regions, and sources of isolation [55,56,57,58,59,60].

Our studies suggest plasticity and stability in the genomes of *S. maltophilia*. Evolutionary studies suggested that mutation could be too slow to allow bacteria to adapt to the dynamic environment. Hence, acquiring new genes is a faster mechanism to enable bacteria to survive in the environment. The open pangenome in *S. maltophilia* [14,15], which is the identification of new gene families with the addition of strains in the population, supports a similar evolutionary trend in the population. The observed trend largely indicates genome plasticity in the *S. maltophilia*. Mobilome played a major role in expanding the accessory genomes of a population, and horizontal gene transfer (HGT) occurred in the pathogen directly from the effect of their surrounding [61]. For instance, most of the identified ICEs and IMEs in this study were found to share a high homology with proteins from *P. aeruginosa*. Multiple studies have proven the direct interactions between *S. maltophilia* and *P. aeruginosa* in the pathogenesis of infected hosts [62,63,64]. The observation could also indicate the potential of genetic acquisition in *S. maltophilia* from bacteria of the same habitat, specifically from *P. aeruginosa*, as predicted in this study. In addition, the observed variations in the GC content and length of these elements may suggest that they have different origins and were acquired at multiple time points during the evolution of these strains. The uneven distribution of ICEs and IMEs in the *S. maltophilia* genomes has also further highlighted that HGT is still ongoing in the population [65]. The presence of various relaxase types and mating pair formation systems suggests a broad range of mechanisms for gene transfer [66]. Many of these elements were situated near tRNA genes, which are known to be common and stable integration sites for MGEs [19], providing a favorable environment for the maintenance of foreign DNA. Secondly, intact phages suggest recent and ongoing active phage infections, while incomplete phages may represent remnants of past phage infections that have undergone mutational decay over time. The similarity in GC content between the phages and their hosts may facilitate more efficient phage–host interactions and integration [67,68]. The two specific prophages conserved across eleven strains suggest these prophages could have infected a common ancestor.

Apart from the genes responsible for mechanisms in successful recombination, such as conjugative elements, recombinase, *attL* and *attR*, these MGEs were predicted to introduce and disseminate virulence and antimicrobial resistance into the population. A study on 78,315 bacterial genomes proposed the existence of old and recent modes of HGT, in which virulence and AMR were involved as recent events in the bacterial population [69]. Although a higher number of VFs were predicted among the clinical strains, our analysis showed that several non-clinical isolates still harbor a high number of VFs, which aligns with the understanding that such genes can be important for general adaptation and survival [70]. We also observed that clinical strains appear more equipped with ARGs than non-clinical strains, which likely reflects their recent encounters with antibiotics in clinical settings. The hypothesis is in accordance with the proposal by Dmitrijeva and colleagues [69].

On top of being able to introduce new gene families into a population, the mobilome is also found to be able to influence the structure of the host’s genome. ISs were predicted to be present abundantly in the *S. maltophilia* population. IS is known as the simplest form of MGE, where it carries only a single transposase for its mobility and repetitive sequences at both ends for integration into the host. Despite its simple structure, ISs are known for being able to regulate the expression of genes [71]. Most of the ISs predicted in *S. maltophilia* were present in the intergenic regions and did not disrupt the structure of the host genes. ISs were reported to be able to mediate chromosomal rearrangements of the host. Other than potentially being involved in the regulation of gene expression, while the overall genome structure of *S. maltophilia* appears to be conserved, the activity of ISs could have also altered the structure of *S. maltophilia*’s genomes. The inversion involving 2,418,035 bp in strain SM 866 is flanked by IS110 family transposases, suggesting these elements, which are known to catalyze site-specific rearrangement [72], may have mediated the event. Similarly, an inversion in strain SJTL3 involved a Tn3 family transposase, a family known for its role in mediating genomic rearrangements [73,74].

In summary, *S. maltophilia* showed plasticity by integrating the mobilomes alongside cargo genes that potentially pose selective advantages and contribute to the diversity of the species. From a different perspective, the presence of mobilomes and rearrangement raised interest in genome stability in *S. maltophilia*. The dynamics of the genome structure and gene content are the two significant components in genome stability [75,76]. Genomic comparisons also indicated that genome stability is more significant in evolution and adaptation of pathogens [77]. Previous studies showed that genomic rearrangements and recombination are able to suppress the association of mutations and weaken the stability of genomes [77,78]. Our analyses indicated recombination was not a significant influence at the backbone (core) of *S. maltophilia*. There are many sites that are still under the influence of linkage disequilibrium (LD). Interestingly, analyses on the gene sets experiencing LD showed strong interactions and mainly involved the regulation of protein expression and maturation. Regulations in both gene expression and maturation have been reported to be highly related to the stability of the host [79,80,81]. On top of that, nucleotide variants were also found to have a direct effect on the stability of molecules [82,83]. We hypothesized that gene expression level and protein folding are also important in *S. maltophilia* adaptation and evolution. Hence, LDs are present among the genes that achieve interactions to perform their functions. However, there are no published works on the prevalence of LD in any functional categories, including the two enriched functional categories predicted in this study.

The view on genome stability in *S. maltophilia* was further supported by the presence of defense genes and toxin–antitoxin. The distribution of defense systems was reported previously by Jdeed and colleagues [84]. With the 20 genomes that were used as a model to study the population of *S. maltophilia*, defense systems were still predicted. Furthermore, there were no consistent features in the defense system separating clinical and non-clinical strains. This could indicate the protection against potential threats brought by foreign molecules into *S. maltophilia* from the diverse environment. Similarly to the previous study [85], there was also no significant difference in the frequencies of toxin–antitoxin (TA) systems between clinical and non-clinical groups. The presence of prevalent systems like PumA/PumB, first identified on a *Pseudomonas aeruginosa* plasmid where it contributes to virulence [86], suggests conserved roles. More importantly, we identified several toxins that were linked to genome defense functions such as abortive infection and prophage maintenance. The MazF toxin, predicted in all twenty genomes, has been associated with cleaving viral RNA to inhibit phage infection [87]. The HigB toxin, found in three clinical and six non-clinical strains, is linked to abortive infection triggered by the recognition of phage proteins [88]. Additionally, the Doc toxin, identified in three clinical and non-clinical strains, is known to be involved in prophage maintenance through post-segregational killing [89]. In view of these functions, the discovery of TA systems within MGEs like prophages and ICEs reflects their function as accessory modules that promote the stabilization and maintenance of these elements [90] and the genome of the host.

## 4. Materials and Methods

### 4.1. Data Acquisition

Complete genomes of *S. maltophilia* were retrieved from the National Center for Biotechnology Information (NCBI) Refseq database. The clinical status of each strain was curated based on its respective publication. Ten genomes of clinical and non-clinical strains were selected for this study.

### 4.2. Prediction of Mobile Genetic Elements (MGEs)

The study focused on integrative and conjugative elements (ICEs), integrative and mobilizable elements (IMEs), prophages, and insertion sequences (ISs). ICEfinder 2.0 [91] was used for ICE and IME prediction. The ICEfinder output provides the number of ICEs and IMEs predicted in a genome, genomic regions, sequences, and annotations. PHASTEST [92] was selected for prophage prediction as it used advanced neural network models, which can identify subtle sequence features that traditional tools might not be able to detect. Additionally, PHASTEST can analyze both complete and draft genomes. The tool was executed in “deep” mode to enhance detection accuracy [93]. The PHASTEST output included prophage classifications as either intact, incomplete, or questionable, along with their genomic regions, sequences, and sequence annotations. Only intact and incomplete prophages were selected for downstream analysis because they are more likely to represent functional or nearly functional prophages [93]. ISs were predicted using ISEScan 1.7.3 [94]. The tool uses Hidden Markov Models (HMMs) to identify and classify IS elements into families based on transposase profile. The ISEScan output details the genomic start and stop coordinates, strand, and family classification for each predicted IS. All the predictions were executed locally on an Ubuntu server.

### 4.3. Functional Annotation

The Comprehensive Antibiotic Resistance Database (CARD) [95] and the Virulence Factor Database (VFDB) [96] were used to identify antibiotic resistance genes and virulence factors within the predicted ICE, IME, and prophage regions. In addition, the predictions of antibiotic resistance genes and virulence factors were also predicted from mobilome-free regions. Prediction of the intrinsic key genes enables evaluation of the potential impact of mobilomes in introducing resistance and virulence advantage into the *S. maltophilia* population.

### 4.4. Genome Rearrangement

Pairwise and multiple genome alignment was performed using Gepard 2.1 [97] and Mauve (build 2015-02-13) [98]. Mauve aligns multiple genomes while preserving synteny blocks and detecting rearrangements across genomes. Gepard evaluates genome conservation in a different algorithm than Mauve, which is based on pairwise sequence collinearity. The draft genomes were reordered according to a reference genome (*S. maltophilia* strain K279a) using the “move contig” function in Mauve prior to pairwise and multiple genome alignment. This step is conducted to reduce the chances of false positive results caused by draft assemblies in genome rearrangement inference.

### 4.5. Prediction of Genome Defense System

TAfinder 2.0 [99] was used to predict type II Toxin-Antitoxin(TA) systems across the *S. maltophilia* genomes. The tool was used to predict well-curated TA gene pairs using sequence similarity and domain architectures. These systems were analyzed to explore their distribution and potential correlations with MGEs and strain pathogenicity. A comprehensive search for a wider array of defense and anti-defense systems was performed using Defense-finder [100].

### 4.6. Recombination and Linkage Disequilibrium (LD) Analyses

Mugsy v1r2.2 [101] was used for multiple genome alignment of all *S. maltophilia* strains. MAF generated from Mugsy was further processed with a custom Python 3.12.0 code to extract the aligned core genome. To ensure reproducibility, this custom Python script has been made publicly available on GitHub at https://github.com/Danny220469/S.-maltophilia-research/blob/main/align_mugsy.py. The resulting alignment files were analyzed using Gubbins 3.4 [102] to predict the rate and effects of recombination over mutation in *S. maltophilia*. The output files included recombination-adjusted phylogenies, per-strain recombination rates, and recombination regions. Site-wise linkage disequilibria were predicted in TASSEL 5.2.94 [103]. The predicted sites were filtered based on the squared coefficient of correlation between the alleles at two loci (r^2^) and the normalized LD coefficient (D′) of 0.8.

### 4.7. Statistical Test

All statistical analyses were conducted using R version 4.3.2 with the rstatix package [104]. Mann–Whitney U tests were performed to evaluate the significance of differences between the clinical (n = 10) and non-clinical (n = 10) groups. For all tests, a *p*-value of < 0.05 was considered statistically significant. In addition to *p*-values, the rank-biserial correlation (r) was calculated as a measure of effect size to determine the magnitude of any observed differences.

## 5. Conclusions

Our study showed active horizontal gene transfers (HGT) in the *S. maltophilia* population, and the event could have been significantly responsible for their phenotypes via integration of cargo genes, e.g., antimicrobial resistances and virulence. On the other hand, recombination was predicted to be minimally impacted on the core genomes with the observations of a low ratio of recombination over mutation rates and the presence of LD sites. Combining the views on the dynamics of accessory and backbone of the genome, we hypothesized the genome stability and plasticity in *S. maltophilia*. The preservation of association among genes that are functionally enriched in protein maturation and regulation remains unclear. However, the analyses could provide insight into the possible mechanisms for adaptation and survival of *S. maltophilia*. The outcomes from the current bioinformatics research would require experimental validations, and we hope that this work can serve as a guide for future *S. maltophilia* research.

## Figures and Tables

**Table 1:** *S. maltophilia* strains used in this study.

Status	Strain (Accession)	Genome Size	CDS	Source	References
Clinical	K279a (AM743169.1)	4,851,126	4412	Human blood, cancer patient	[35]
Clinical	D457 (HE798556.1)	4,769,156	4313	Clinical isolate	[36]
Clinical	MER1 (CP049368.1)	4,547,296	4047	Hospital wastewater	[37]
Clinical	CYZ (CP101622.1)	4,517,685	4077	Sputum, immunocompromised patient	[38]
Clinical	SM 866 (NZ_CP031058.1)	5,086,181	4692	Tertiary care unit of India	[39]
Clinical	ISMMS2R (CP011306.1)	4,509,724	4016	Bacteremic patient	[40]
Clinical	SCAID WND1-2022 (370) (NZ_CP102942.1)	4,880,425	4387	Intensive care unit	[41]
Clinical	WJ66 (AZRF01000001.1)	4,642,894	4196	Human blood, chemotherapy patient	[42]
Clinical	JUNP497 (DAOKEV010000001.1)	4,853,141	4444	Patients at two university hospitals in Nepal	[43]
Clinical	JUNP350 (DAOKEW010000001.1)	4,202,951	3707	Patients at two university hospitals in Nepal	[43]
Non-clinical	1800 (NZ_OU943334.1)	4,837,108	4433	Effluent of an industrial oil refinery in Algeria	[44]
Non-clinical	W18 (CP028358.1)	4,738,432	4286	Crude oil-contaminated soil	[45]
Non-clinical	SJTL3 (CP029773.1)	4,891,004	4367	Wastewater	[46]
Non-clinical	WGB211 (NZ_CP090418.1)	4,913,676	4467	Shale oil in the Ordos Basin	[47]
Non-clinical	GYH (NZ_CP090423.1)	4,791,742	4324	Environmental isolate	[48]
Non-clinical	KMM 349 (NZ_AP021867.1)	4,578,300	4043	Deep-sea invertebrates	[49]
Non-clinical	503 (CP136922.1)	4,857,915	4359	Campus soil	[50]
Non-clinical	OUC_Est10 (NZ_CP015612.1)	4,668,743	4182	Environmental isolate	[51]
Non-clinical	B418 (JSXG01000001.1)	4,688,249	4093	Barley rhizosphere	[52]
Non-clinical	Col1 (CP077679.1)	4,458,565	3982	Soil	[53]

**Table 2:** Virulence factors predicted within the MGEs of *S. maltophilia* strains.

MGE	Host Strain(s)	Status	Putative Virulence Factor(s)	Putative Function Category
ICE	D457	Clinical	*phoR/mprA*, *bfmR/bfmS*, *allS*	Regulation
			*adeG*, *adeH*, *acrA*	Biofilm/Efflux
	SM 866	Clinical	*adeH*, *acrB*	Biofilm/Efflux
			*fbpC*	Metabolism (Iron Transport)
	1800, WGB211	Non-Clinical	*adeF*, *adeG*, *mtrE*	Biofilm/Efflux
			*ctpV*	Metabolism (Copper Export)
	1800	Non-Clinical	*allS*	Metabolism (Allantoin Use)
	K279a	Clinical	siderophore transporter	Metabolism (Iron Transport)
	503	Non-Clinical	*fslD*	Metabolism (Iron Transport)
IME	K279a, SJTL3	Clinical and Non-Clinical	*upaG/ehaG*, *tagX*	Adherence, T6SS
	503	Non-Clinical	*upaG/ehaG*, *tagX*	Adherence, T6SS
	SJTL3	Non-Clinical	*csuD*	Biofilm (Pilus Assembly)
Phage	WJ66, JUNP497, SCAID WND1-2022	Clinical	*clpP*	Stress Survival
	B418	Non-Clinical	*clpP*	Stress Survival
	W18	Non-Clinical	*recN*	Stress Survival (DNA Repair)
	1800, W18, WGB211	Non-Clinical	*mshM*	Adherence (MSHA Pili)
	1800	Non-Clinical	*sigA/rpoV*	Regulation
	SM 866, W18	Clinical and Non-Clinical	*ptxR*	Regulation (Pyoverdine)
	K279a	Clinical	*adhD*	Immune Modulation
	MER1	Clinical	*pta*	Effector Delivery
	D457	Clinical	*pkn5*	Effector Delivery
	JUNP497	Clinical	*guaB*	Metabolism

**Table 3:** Enrichment Analyses on LD Genes.

Cluster	PPI Enrichment *p*-Value:	BP	MF	CC	Reactome
1	1.48 × 10^−11^	Regulation of translation	NADH dehydrogenase activity	Oxidoreductase complex	Infection with Mtb
3	1.48 × 10^−11^	Regulation of translation	NADH dehydrogenase activity	Oxidoreductase complex	Infection with Mtb
5	1.48 × 10^−11^	Regulation of translation	NADH dehydrogenase activity	Oxidoreductase complex	Infection with Mtb
642	1.48 × 10^−11^	Regulation of translation	NADH dehydrogenase activity	Oxidoreductase complex	Infection with Mtb
912	1.48 × 10^−11^	Regulation of translation	NADH dehydrogenase activity	Oxidoreductase complex	Infection with Mtb
1014	1.48 × 10^−11^	Regulation of translation	NADH dehydrogenase activity	Oxidoreductase complex	Infection with Mtb
1665	4.03 × 10^−10^	Cell septum assembly	Purine ribonucleoside triphosphate binding	Intracellular anatomical structure	-
1907	1.48 × 10^−11^	Regulation of translation	NADH dehydrogenase activity	Oxidoreductase complex	Infection with Mtb
2584	5.69 × 10^−05^	Ribonucleotide metabolic process	-	Catalytic complex	-
320	7.75 × 10^−08^	Gene expression	Quinone binding	Intracellular anatomical structure	-
2342	1.48 × 10^−11^	Regulation of translation	NADH dehydrogenase activity	Oxidoreductase complex	Infection with Mtb
2663	1.48 × 10^−11^	Regulation of translation	NADH dehydrogenase activity	Oxidoreductase complex	Infection with Mtb
512	1.09 × 10^−10^	Gene expression	-	Intracellular anatomical structure	-
1640	1.0 × 10^−16^	Translation	Ion binding	Intracellular anatomical structure	-
2157	2.56 × 10^−06^	Gene expression	Heterocyclic compound binding	Intracellular anatomical structure	-
827	7.75 × 10^−08^	Cellular respiration	Quinone binding	Intracellular anatomical structure	-
967	2.56 × 10^−06^	Gene expression	Heterocyclic compound binding	Intracellular anatomical structure	-
616	4.98 × 10^−05^	Translation	Ion binding	Intracellular anatomical structure	-
172	0.000198	Carboxylic acid metabolic process	Carbon-nitrogen lyase activity	Oxoglutarate dehydrogenase complex	Latent infection—Other responses of Mtb to phagocytosis
2318	1.48 × 10^−11^	Regulation of translation	NADH dehydrogenase activity	Oxidoreductase complex	Infection with Mtb

## Data Availability

The data that support the findings of this study are available in the Supplementary Materials of this article. The Python script used for the analysis is available in a GitHub repository (https://github.com/Danny220469/S.-maltophilia-research).

## Supplementary Materials

The following supporting information can be downloaded at: https://www.mdpi.com/article/10.3390/ijms262110477/s1.
