# Supplementary material for: Comparative Analyses Suggest Genome Stability and Plasticity in Stenotrophomonas maltophilia
Source: Int J Mol Sci. 2025 Oct 28;26(21):10477. doi: 10.3390/ijms262110477 (PMC12607529; doi:10.3390/ijms262110477)
Supplement: Supplementary file 1 [file ijms-26-10477-s001.zip › Supplementary Figures.pdf]

Supplementary Informations

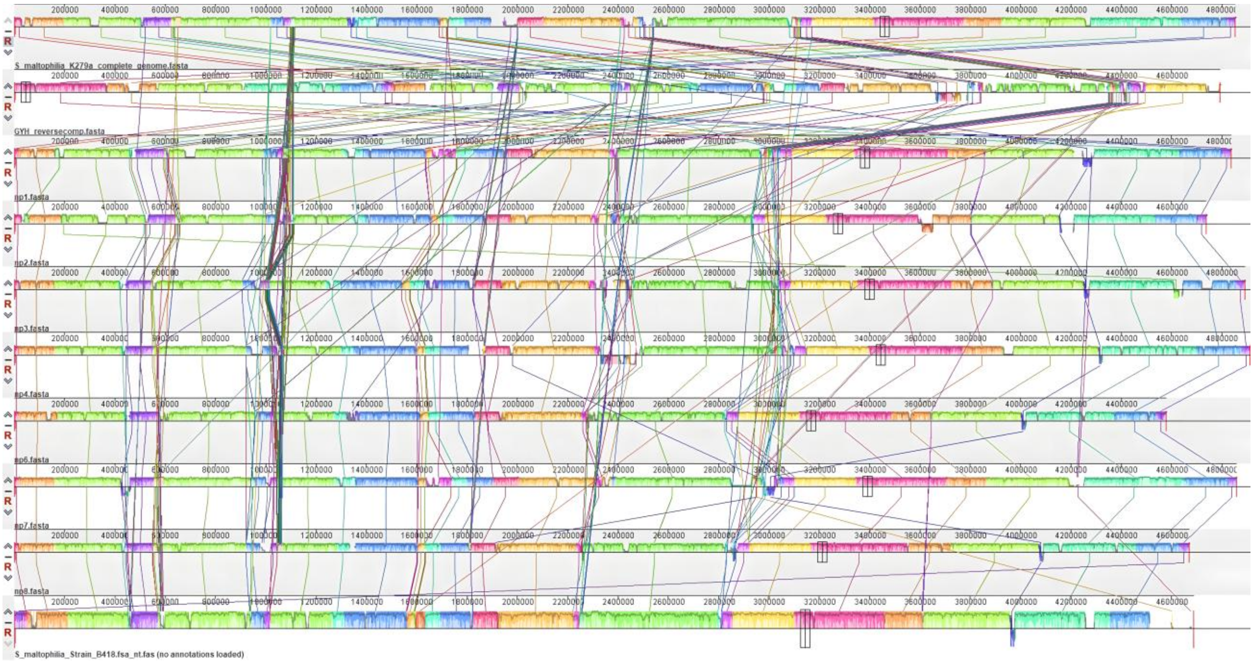

Figure S1. Mauve alignment of non-clinical *S. maltophilia* strains showing conserved genomic architecture. The alignment compares selected non-clinical isolates to the *S. maltophilia* K279a reference (top). Homologous blocks are linked by lines, illustrating a high degree of synteny and fewer large-scale rearrangements compared to the clinical isolates.

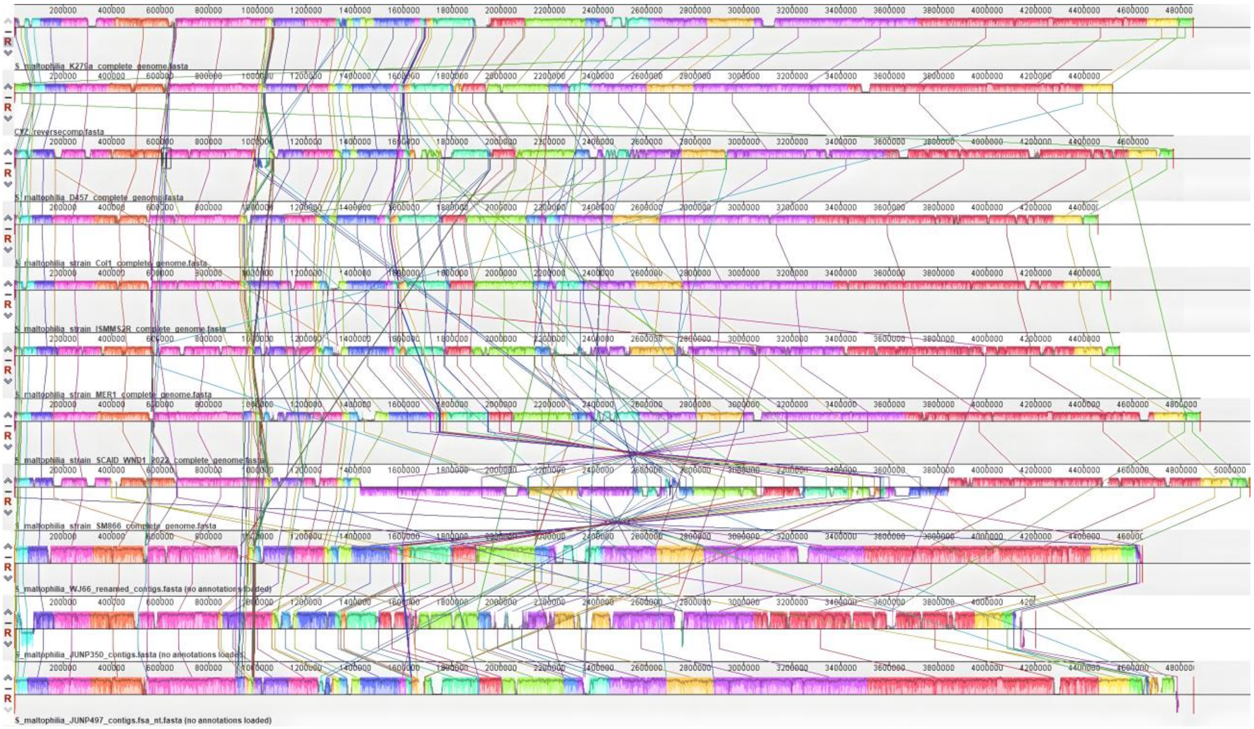

Figure S2. Multiple genome alignment predicted genomic rearrangements and conserved regions among *S. maltophilia* clinical isolates. The Mauve alignment illustrates homologous regions (Local Collinear Blocks, LCBs) shared across the genomes of selected clinical *S. maltophilia* strains. Blocks above the center line indicate regions in the forward orientation, while blocks below the center line are inverted relative to the reference genome (*S. maltophilia* K279a). Lines connecting the blocks highlight synteny, with crossing lines indicating inversions or translocations.

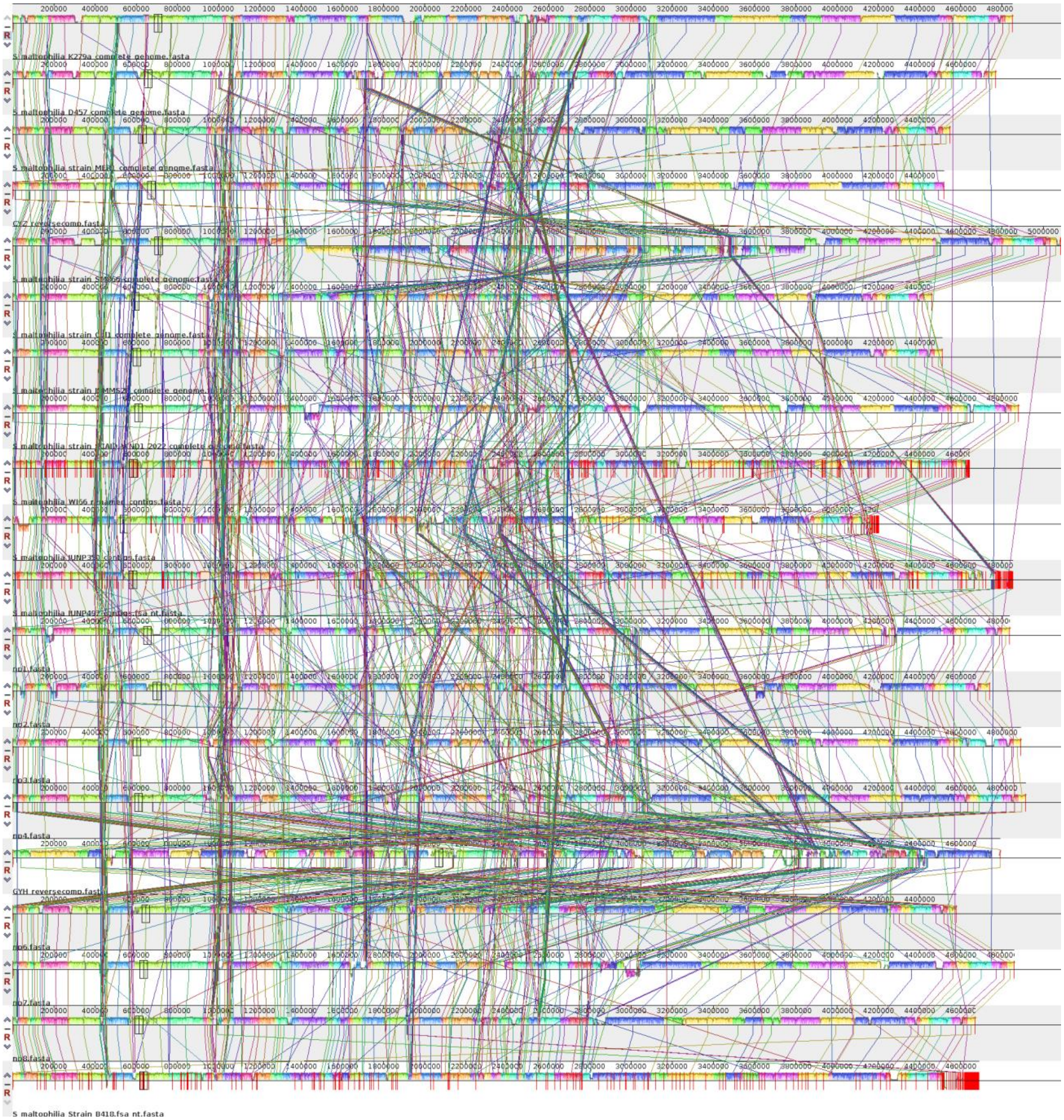

Figure S3. Whole-genome alignment of the complete cohort of 20 *S. maltophilia* strains.

The Mauve alignment displays the genomic architecture across all clinical and non-clinical isolates, with *S. maltophilia* K279a as the top reference. Homologous regions (colored blocks) are connected by lines, illustrating both large-scale synteny and significant genomic rearrangements. Blocks positioned below the center line represent inversions relative to the reference, and crisscrossing lines indicate complex translocations or inversions, highlighting the genomic plasticity across the species.

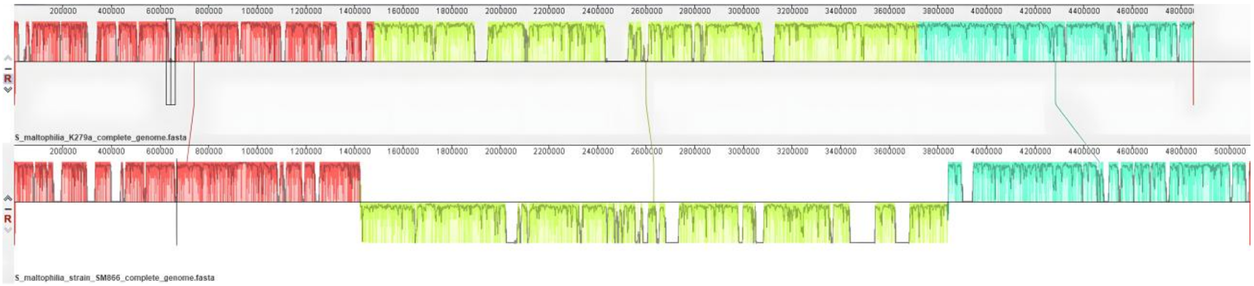

Figure S4. A large-scale genomic inversion in *S. maltophilia* strain SM 866. The Mauve alignment of the SM 866 genome against the K279a reference reveals a single, large inversion of approximately 2.4 Mbp. The inverted block, shown below the center line, is flanked by insertion sequences, suggesting a potential mechanism for the rearrangement.

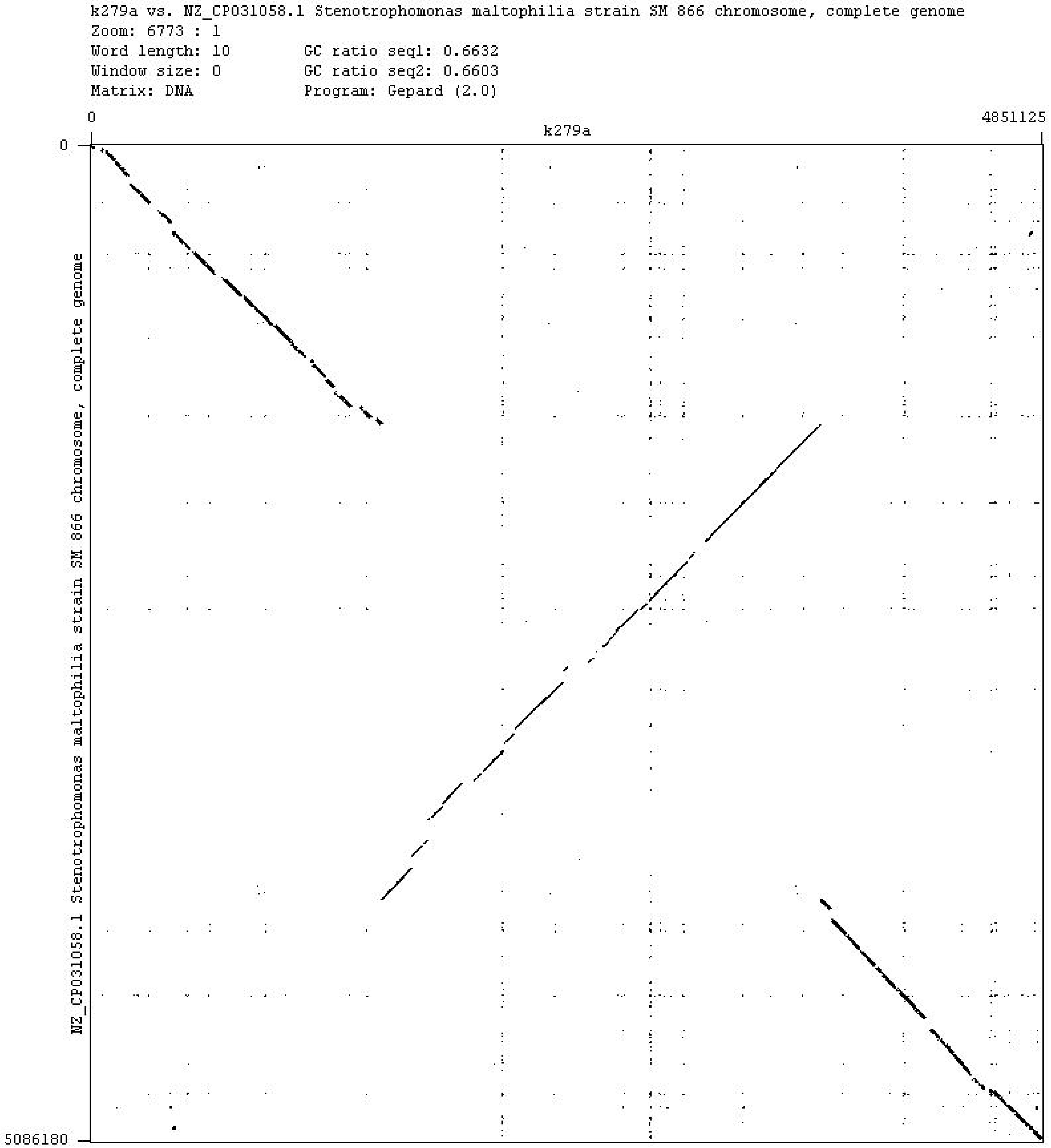

Figure S5. Gepard dot plot analysis supporting the ~2.4 Mbp genomic inversion in *S. maltophilia* strain SM 866. The plot compares the genome of SM 866 (Y-axis) against the K279a reference (X-axis). The two short lines along the main diagonal represent regions of conserved synteny. The prominent anti-diagonal line, running from top-left to bottom-right, visually confirms the presence of a single, large-scale inversion of approximately 2,418,035 bp in strain SM 866.
